# Protective effect of interferon type I on barrier function of human airway epithelium during rhinovirus infections in vitro

**DOI:** 10.1038/s41598-024-82516-2

**Published:** 2024-12-16

**Authors:** Helena Boland, Adrian Endres, Ralf Kinscherf, Ralf Schubert, Beate Wilhelm, Hans Schwarzbach, Danny Jonigk, Peter Braubach, Gernot Rohde, Carla Bellinghausen

**Affiliations:** 1 Department of Respiratory Medicine and Allergology, Medical Clinic 1, University Hospital Frankfurt, Goethe University, Frankfurt am Main, Germany; 2 Department of Medical Cell Biology, Institute for Anatomy and Cell Biology, University of Marburg, Marburg, Germany; 3 Division of Pediatrics, Pulmonology, Allergology, Infectious Diseases and Gastroenterology, Children’s Hospital, University Hospital Frankfurt, Goethe University, Frankfurt am Main, Germany; 4 Institute for Pathology, UKA University Medical Center RWTH University Aachen, Aachen, Germany; 5 Institute of Pathology, Hannover Medical School, Hanover, Germany; 6 German Center for Lung Research (DZL), Biomedical Research in End-stage and Obstructive Lung Disease Hannover (BREATH), Hannover, Germany

**Keywords:** Cell biology, Immunology, Virology

## Abstract

The airway epithelium provides a crucial barrier against infection with respiratory pathogens. This barrier can be impaired following viral infection, paving the way for bacterial superinfections. Type I interferons (IFNs) are important antiviral mediators, and inhaled formulations of these glycoproteins are considered a potential approach for the treatment of respiratory viral infections. To investigate if type I IFNs can also protect against virus-induced epithelial barrier dysfunction, differentiated primary bronchial epithelial cells were pre-treated with IFN-β1a and subsequently infected with human rhinovirus (HRV) for 24 to 72h. Moreover, to functionally assess the effects of IFN-β1a pre-treatment on barrier integrity, we conducted co-infection experiments, in which cells were initially infected with HRV, and superinfected with *Streptococcus pneumoniae* 24 to 72 h later. In untreated cells, HRV infection significantly damaged ZO-1 positive tight junctions and cilia, and transiently increased permeability, whereas the barrier of cultures pre-treated with IFN-β1a remained intact. In co-infection experiments, bacteria were able to penetrate deeper into the cell layers of HRV-infected cultures than into those of uninfected cells. IFN-β1a pre-treatment abrogated virus-induced damage to the epithelial barrier. Taken together, these data demonstrate a beneficial effect of IFN-β in protecting epithelial barrier function in addition to its antiviral effects.

## Introduction

The airway epithelium constitutes an important physical barrier of the human body towards the environment, and thus provides nonspecific, mechanical protection against pathogens, pollutants, and other harmful agents^1,2^. Its pseudostratified structure consists of different epithelial cell types, including basal cells, mucus-producing goblet cells, and ciliated cells. Collectively, they protect underlying tissues by means of mucociliary clearance, recognition of conserved pathogen structures, production of antibacterial and antiviral mediators, and by maintaining tight intercellular connections^3–5^.

Cell–cell connections in the airway epithelium include gap junctions, desmosomes, adherence junctions and tight junctions (TJs)^6,7^. TJs are located at the apical-lateral border, forming a ring around epithelial cells, thereby delimiting the apical from the basal side. Their main function is the regulation of the selective flow of ions and solutes through the paracellular space and prevention of paracellular migration of pathogens^6,8^. TJ are multiprotein compound complexes composed of transmembrane proteins (like claudin), junctional adhesion molecules (occludin and tricellulin), and associated cytoplasmic plaque proteins (zonula occludens (ZO)-1, 2 and 3)^9–12^. ZO proteins are able to build a direct connection to other TJ-proteins and the actin cytoskeleton via protein–protein interactions^13–15^.

As an important physical barrier, airway epithelial cells also play a pivotal role in the recognition of pathogens, and in the recruitment and activation of innate immune cells. Using pattern recognition receptors (PRRs), epithelial cells recognize conserved structural motifs and molecules, so-called pathogen-associated molecular patterns (PAMPs). Recognition of PAMPs triggers the activation of innate immune cascades, leading to the production of cytokines and chemokines, as well as production of antiviral and antibacterial mediators. Among the key mediators involved in the defense against respiratory viruses are interferons (IFNs)^1,16,17^. Based on their receptor utilization, IFNs are divided into three groups (IFN type I-III)^18^. While IFN γ, the only type II IFN, is mainly produced in natural killer cells and T cells^19^, type I and III IFNs are predominantly produced by epithelial cells to restrict viral replication, and establish an antiviral state in directly affected and neighboring cells^20^. By binding to their respective receptors, type I and III IFNs activate the expression of IFN-stimulated genes (ISGs) through activation of the janus kinase (JAK)—signal transducer and activator of transcription (STAT) signaling cascade, and subsequent phosphorylation and translocation of the ISG factor 3 (ISGF3) complex to the cell nucleus^21–23^.

Local application of exogenous type I IFNs in the respiratory tract has been suggested as antiviral treatment for infections with respiratory viruses^24,25^. Human rhinoviruses (HRV) are the most common cause of upper respiratory tract infections and can be detected all year round with peaks in early fall^26^. While infections are usually restricted to the upper respiratory tract and self-limiting in otherwise healthy individuals, they can lead to severe lower respiratory tract symptoms in people with chronic respiratory diseases and aggravate their disease.

Infections with HRV and other respiratory viruses can increase the permeability of the airway epithelial barrier as well as its mucus production, and can affect the number of cilia^27–29^. Moreover, they disturb barrier function and lead to a degradation of tight and adherence junctions^27,28,30,31^. Additionally, virus-induced disruption of the epithelial barrier and associated increased permeability may facilitate invasion of bacteria and thus, secondary bacterial infections^30,32^.

We and others have shown* in vitro* that treatment with exogenous IFN-β (type I IFN) or IFN-λ1 (type III IFN) induces a long-lasting antiviral state in respiratory epithelial cells and reduces infection with viruses like HRV^33–35^. Clinical studies investigating the use of inhaled type I IFN for treatment of virus-associated exacerbations of chronic obstructive pulmonary disease and asthma, as well as for SARS-CoV2 infection are underway^36,37^. Whether or not IFNs can also protect the airway epithelium from virus-induced barrier dysfunction and invasive bacterial infection is so far unclear. In this project, we aimed to investigate the effects of IFN-β pre-treatment on the barrier function of respiratory epithelial cells during viral infection.

## Methods

### Cell culture

Primary bronchial epithelial cells (pBECs) were isolated from lung explant tissue of four cystic fibrosis patients. Tissue was provided by the Lung Tissue Collection of the Department of Pathology, Hannover Medical School. Use of this tissue was approved by the institutional ethics review board of Hannover Medical School (Reference 2700–2015).

Basal cell populations were isolated as previously described^38^, with minor modifications^39^. Epithelial origin of the isolated cells was confirmed by immunofluorescence. Cells stained positive for cytokeratin 5 (rabbit anti-human cytokeratin 5, 1 µg/mL, 1 h at RT, Biolegend, San Diego) and p63 (rabbit anti-human p63, 1 µg/mL, 1 h at RT, Abcam, Cambridge), and negative for fibroblast-reactive antibody TE-7 (mouse anti-human fibroblast antigen, clone TE7, 2 µg/mL, 1 h at RT, Merck, Darmstadt).

For each experiment, cryopreserved pBECs were thawed, cells were expanded and passaged once in tissue culture flasks pre-coated with 10 µg/mL fibronectin (VWR, Radnor), 30 µg/mL collagen (PureCol, Sigma Aldrich, Taufkirchen) and 10 µg/mL bovine serum albumin (BSA, Sigma Aldrich, Taufkirchen) in PBS, using PneumaCult Ex Plus medium including supplements (StemCell Technologies, Vancouver). Cells were then seeded at 4 × 10^5^ cells/insert into the apical chamber of transwell inserts (TC-Inserts 12 Well PET 0.4 µm, Sarstedt, Nümbrecht) in a 12-well plate, which were pre-coated with the same coating solution as tissue culture flasks. Cells were initially cultured submerged with PneumaCult Ex medium on the apical and basal compartment of the insert, and medium was replaced every two to three days. When cell layers were fully confluent, differentiation as air–liquid-interface (ALI) culture was initiated by removing the medium on the apical side of the cell layer, and replacing the medium on the basal side with PneumaCult ALI medium including supplements (StemCell Technologies, Vancouver), antibiotic/antimycotic solution (Sigma Aldrich, Taufkirchen), and MycoZap Plus PR (Lonza, Basel). Every two to three days, until cells were fully differentiated, cell layers were rinsed three times with PBS and medium was refreshed. Differentiation was achieved within three to five weeks after initiation of ALI culture and was confirmed by increased transepithelial electric resistance (TEER), mucus production, and microscopically visible ciliary movement.

### Virus and bacteria

HRV 16 (ATCC, Manassas) was propagated in HeLa Ohio cells (Sigma Aldrich, St. Louis) and cultured as described previously^39^. The 50% Tissue Culture Infectious Dose (TCID_50_) of virus stocks was determined on HeLa Ohio cells as described below.

*Streptococcus pneumoniae* Danish designation 19F (ATCC, Manassas) was grown overnight on Columbia Agar plus Sheep Blood ‘Plus’ plates (Thermo Fisher Scientific, Waltham). For achieving an equal bacterial inoculum in every experiment, bacterial suspensions were prepared in PBS and adjusted to an OD_600_ of 1.3. These suspensions were then further diluted, using an experimentally determined dilution factor to achieve 2 × 10^7^ colony-forming units (CFU) per 300 µL.

### Pre-treatment of ALI cultures with IFN-β1a and infection protocol

Prior to IFN-β1a pre-treatment, apical sides of ALI cultures were washed three times with PBS and basal medium was refreshed. Per insert, 1600 ng IFN-β1a (Peprotech, Rocky Hill) in 100 µl PBS + antibiotic/antimycotic solution were added to the apical side of the cells. After 24 h incubation at 37 °C and 5% CO_2_, and prior to viral infection, IFN-β1a was removed, basal medium was refreshed, and cells were rinsed with PBS.

Subsequently, cells were infected from the apical side with 100 μL of HRV 16 diluted in PBS including antibiotic/antimycotic solution, to achieve a multiplicity of infection (MOI) of 1. After Incubation at 33 °C and 5% CO_2_ for 2 h, the virus inoculum was removed from the apical compartment. Cells were then re-stimulated with 1000 ng IFN-β1a diluted in 100 µL PBS including antibiotic/antimycotic solution and incubated at 33 °C and 5% CO_2_ for 24 h to 72 h.

For co-infections, antibiotics were omitted from the culture media seven days prior to bacterial infection. To simulate a viral-bacterial co-infection, cells were infected with *S. pneumoniae* at the indicated time points after HRV 16 infection. Cell layers were washed twice with PBS before infecting the cells, and 2 × 10^7^ CFU of *S. pneumoniae* diluted in 300 µL PBS were added to the apical side of the insert (MOI 10). Afterwards cells were incubated for 8 h at 37 °C and 5% CO_2_.

A schematic overview of the infection and stimulation protocol is depicted in figure S1.

### Measurement of epithelial barrier function

Prior to IFN stimulation as well as before and after 24 h to 72 h HRV-infection, TEER was measured as an indicator of epithelial barrier integrity (Millicell ERS-2 instrument, MerckMillipore, Burlington). Cells were washed from the apical side to remove mucus, and basal medium was replaced with PBS before recording the TEER (R _insert_). Resistance of an insert without cells (R _blank_) was measured in parallel. TEER values corrected for blank measurement and surface area of the insert (A _insert_) were calculated as R _corrected_ = R _insert_ – R _blank_ * A _insert_.

Additionally, permeability of the cell layer was assessed using FITC-labeled dextran. At the indicated time points, 100 µL FITC-labeled dextran (5 mg/mL, diluted in PBS, average molecular weight 3–5 kDa, Sigma Aldrich, St. Louis) were added to the apical side of ALI cultures. After 1 h of incubation, fluorescence was measured in basal medium (EnVision Multimode Plate Reader, PerkinElmer, Waltham).

### Reverse transcription quantitative PCR (RT qPCR)

Gene expression and viral RNA were quantified by RT qPCR. Therefore, total RNA was isolated using the peqGOLD Total RNA Kit (VWR, Radnor), and 200 ng RNA were reverse transcribed using the iScript gDNA Clear cDNA Synthesis Kit (Bio-Rad Laboratories, Hercules) according to the manufacturers’ instructions. cDNA was then diluted 1:10. qPCR was performed using Sso Advanced Universal Probes Mastermix (Bio-Rad Laboratories, Hercules) on a Bio-Rad CFX Opus 96 Real-time PCR system (enzyme activation: 2 min, 95 °C; denaturation: 15 s, 95 °C; annealing/elongation: 1 min, 60 °C; denaturation and annealing/elongation repeated 40 times (41 cycles)). Primer and probe sequences are listed in table S1.

For calculation of fold changes, the 2^-∆∆CT^ method was used^40^. Fold changes were calculated relative to uninfected control cells and using GAPDH for internal normalization. Quantification of viral RNA copies was carried out based on a standard curve derived from synthetic double-stranded DNA corresponding to the target region of the RT qPCR assay for HRV (gBlocks, IDT, Coralville).

### 50 % Tissue culture infectious dose (TCID_50_)

The infectious dose of HRV 16 stocks and experimental samples was assessed by determining the TCID_50_. Virus stocks or apical wash fluid of HRV-infected cells were diluted (10^–2^ to 10^–10^) in Gibco DMEM (Thermo Fisher Scientific, Waltham) + 2% fetal calf serum (Sigma Aldrich, Taufkirchen). Per dilution, eight wells of confluent HeLa Ohio cells (seeded at 2 × 10^4^ cells in 100 µL / well of 96 well plate and incubated overnight) were infected and incubated at 33 °C and 5% CO_2_ for 2 h. After this initial attachment period, the medium was replaced with virus-free medium. Cells were incubated for 5 days at 33 °C and 5% CO_2,_ and cultures were then visually inspected for cytopathic effect. The TCID_50_ was calculated using the Spearman-Karber Formula^41^.

### Cell viability analyses

As an indicator for cytotoxicity, lactate dehydrogenase (LDH) levels were determined in apical wash fluid using the CyQUANT LDH Cytotoxicity Assay (Invitrogen, Waltham) according to the manufacturer’s instructions. Values show the mean of blank-corrected measured optical density at 458 nm.

Additionally, cell death was determined by flow cytometry using the LIVE/DEAD Fixable Blue Dead Cell Stain Kit, for UV excitation (Invitrogen, Waltham) according to manufacturer’s instructions. In brief, cells were washed with PBS, harvested with trypsin/EDTA (Sigma Aldrich, Taufkirchen), stained with LIVE/DEAD Fixable Blue Dead Cell Stain Kit and subsequently fixed for 15 min with 4% (w/v) formaldehyde. The percentages of live and dead cells were then quantified on a BD FACSCanto II Flow Cytometry System (BD, Franklin Lakes). Stained polystyrene beads (ArC Amine Reactive Compensation Bead Kit, Invitrogen, Waltham) were used for spectral compensation and optimization of settings.

### Immunofluorescence

Cells were washed with PBS and fixed for 15 min with 4% (w/v) formaldehyde. Cells were then rinsed with PBS, permeabilized for 15 min with 0.25% Triton X-100 in PBS, washed again with PBS and unspecific binding sites were blocked with 1% BSA in PBS for 1 h. Afterwards, cells were incubated with primary antibodies protected from light exposure, all diluted in 1% BSA in PBS. For staining ZO-1 and basal cells, cells were incubated with mouse anti-human ZO-1 (FITC-conjugated, 2.5 µg/mL, at room temperature, Thermo Fisher Scientific, Waltham) for 2 h, and an additional 1 h with rabbit anti-human p63 (1 µg/mL, at room temperature, Abcam, Cambridge). For co-staining of *S. pneumoniae*, cells were incubated with rabbit anti-*S. pneumoniae* (8 µg/mL, 1 h at room temperature, Bio-Rad Laboratories, Hercules) instead of the anti-p63 antibody. Cells were washed again after incubation with the primary antibody, and before adding conjugated fluorescent secondary antibody AlexaFluor555 Goat α-rabbit (2 µg/mL diluted in 1% BSA in PBS, 1 h at room temperature). Isotype control stainings were performed using Mouse IgG2 kappa Isotype Control (FITC conjugated, Thermo Fisher Scientific, Waltham, for anti-ZO-1 antibody), Rabbit IgG monoclonal EPR25A Isotype control (abcam, Cambridge (UK), for anti-p63 antibody) or Rabbit IgG Isotype Control (polyclonal, Thermo Fisher Scientific, Waltham, for anti-*S. pneumoniae* antibody) instead of the respective primary antibody. Cells were rinsed again with PBS, transwell membranes were detached from inserts using a scalpel and mounted on slides with ProLong Diamond Antifade with DAPI (Invitrogen, Waltham). Confocal laser scan microscopy was performed using a Leica DMI6000 B Fully Automated Inverted Research Microscope (× 63 immersion oil objective) and Leica LAS AF software.

### Image processing and analysis

ZO-1 area and depth of penetration of *S. pneumoniae* into the cell layer were analyzed based on Z stacks and orthogonal views generated from CLSM images using FIJI ImageJ2^42^. Z stack projections were generated using the maximum intensity setting of the respective ImageJ2 function. Contrast stretching was performed for each channel of composite projection images or orthogonal views separately and applied to the whole image uniformly. Insets for magnification and figures showing immunofluorescence images were created using the QuickFigures Plugin for ImageJ^43^.

For analyzing ZO-1 area, projections for the ZO-1 channel were generated for stacks from each time point and experiment. Contiguous staining areas of up to 1.5µm^2^ in these images were then quantified using an ImageJ macro, using the whole image as input. Quantification data are based on four independent experiments using cells from different donors for each experiment.

For assessing the depth of penetration of bacteria into the cell layer, five orthogonal views were created from two Z stacks for each experimental condition and experiment. Afterwards, the height of the cell layer was determined at three points in the orthogonal view using FIJI ImageJ2. The values where then used to calculate the mean height of the cell layer. The distance of each visible, individual bacterial dot to the basal end of the cell layer was measured and the average of these values (*distance bacteria*) and the mean height of the cell layer were then used to calculate how far the bacteria had penetrated into the cell layer, expressed as % of the average cell layer depth ((1-*distance bacteria*/ mean height of the cell layer)*100).

### Electron microscopy

Cells were washed with PBS from the apical side and fixed in 2.5% (v/v) glutaraldehyde (Serva, Heidelberg), 1.25% (w/v) formaldehyde, 2 mM picric acid (in 0.1 M cacodylate buffer, pH 7.0) and further treated with 1% (w/v) osmium tetroxide. Transwell membranes were detached from inserts using a scalpel and afterwards cells were dehydrated with ethanol/propylene oxide and embedded in glycid-ether 100 (EPON 812, Carl Roth, Karlsruhe). Samples were cut with a Reichert Ultracut S ultramicrotome (Leica Microsystems, Wetzlar) using a diamond knife. Ultrathin sections (70–80 nm) were placed on copper grids, dried overnight and contrasted with 4% uranyl acetate and lead citrate. Imaging was performed with a Zeiss EM 10 C TEM (Carl Zeiss GmbH, Jena) and pictures were obtained with ImageSP System (SYSPROG, Minsk).

### Statistical analysis

Statistical analysis was carried out using data from four independent experiments with identical experimental conditions. Each independent experiment contained technical duplicates per condition, of which the arithmetic mean was used for analysis. Data in figures and text are shown as untransformed values (mean + /- standard deviation (SD)) unless otherwise indicated.

For comparison of viral loads between two treatment groups, values were logarithmically transformed to base 10 and a paired t-test was carried out. Statistical significance for comparing several groups, for example values of a time series, was tested with a two-way or three-way analysis of variance (ANOVA), followed by a Tukey’s multiple comparison test. The significance level was set at α = 0.05.

## Results

### IFN-β1a pre-treatment has antiviral effects and decreases HRV-induced cytopathic effect

**Fig. 1:**
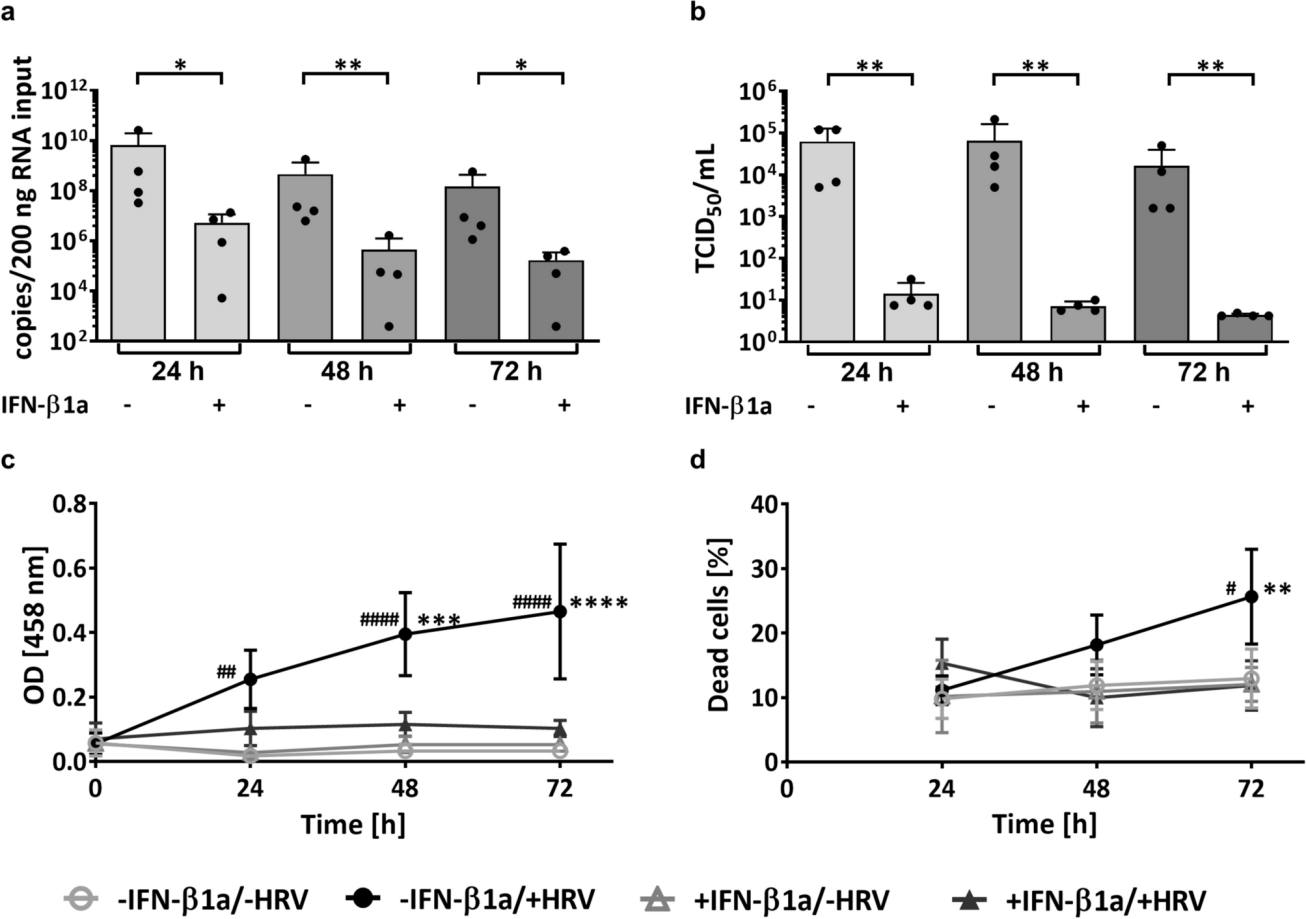
Viral load and cell viability of differentiated pBECs in ALI culture after 24 h IFN-β1a pre-treatment and HRV 16 infection. (**a**) Viral RNA copies at indicated timepoints measured by RT qPCR and (**b**) infectious particles determined as TCID_50_. Cell viability was determined by (**c**) lactate dehydrogenase (LDH) assay and (**d**) LIVE/DEAD assay. n = 4, data represent mean ± SD, * -IFN-β1a/ + HRV vs. + IFN-β1a/ + HRV, # -IFN-β1a/ + HRV vs. -IFN-β1a/-HRV; */# p ≤ 0.05; **/## p ≤ 0.01; ***/### p ≤ 0.001; ****/#### p ≤ 0.0001. (**a**) & (**b**): log10 transformed data, paired t-test, two-tailed, per time point. (**c**) & (**d**): Three-Way ANOVA, Tukey’s Multiple Comparison Test, matching factor: time after infection.

Exogenous IFN-β has previously been shown to elicit potent antiviral effects in different respiratory viral infection models^33,35,44^. To confirm these findings in the model used in this study, differentiated pBECs in ALI culture were stimulated for 24 h with IFN-β1a and subsequently infected with HRV 16 for up to 72 h. IFN-β1a pre-treatment reduced the number of viral RNA copies on average by up to 99.8% (e.g., 24 h: -IFN-β1a/ + HRV 6.63 × 10^9^ ± 1.28 × 10^9^ HRV copy number /200 ng RNA vs. + IFN-β1a/ + HRV 5.29 × 10^6^ ± 6.18 × 10^6^ HRV copy number /200 ng RNA, p = 0.021) (Fig. 1a). Likewise, the number of infectious particles as determined by TCID_50_ was significantly reduced in IFN-β1a pre-treated cells (Fig. 1b).

In the absence of IFN-β1a pre-treatment, HRV 16 infection was associated with significantly higher LDH release when compared to IFN-β1a pre-treated, infected cells, or untreated, uninfected cells, indicating that HRV 16 infection affected cell viability in this model (e.g., LDH; 24 h: -IFN-β1a/ + HRV OD_458_ 0.25 ± 0.09 vs. -IFN-β1a/-HRV OD_458_ 0.02 ± 0.02, p = 0.002) (Fig. 1c).

Similar, albeit less pronounced, results were obtained when measuring the percentage of dead cells by flow cytometry (LIVE/DEAD assay). The percentage of dead cells was significantly higher after 72 h of HRV 16 infection in untreated, infected cells when compared to untreated, uninfected control cells (-IFN-β1a/ + HRV 25.65 ± 7.36% dead cells vs. -IFN-β1a/-HRV 12.95 ± 4.58% dead cells, p = 0.012) and IFN-β1a pre-treated, HRV 16-infected cells (-IFN-β1a/ + HRV 25.65 ± 7.36% dead cells vs. + IFN-β1a/ + HRV 11.86 ± 3.85% dead cells, p = 0.005) (Fig. 1d). At the earlier time points, there were no statistically significant differences between the treatment conditions.

To assess epithelial response to infection, cytokine concentrations were measured in basal media. As expected, IL-8, IL-6, IP-10 and TNF-α concentrations in basal media of HRV 16-infected and untreated cells increased compared to the untreated, uninfected control cells. With the exception of IP-10, concentrations of all other cytokines measured were lower in cells which received IFN-β1a pre-treatment prior to and during viral infection (figure S2a-d).

### Pre-treatment with IFN-β1a protects pBECs in ALI culture from HRV 16-induced damage of TJ complexes

**Fig. 2:**
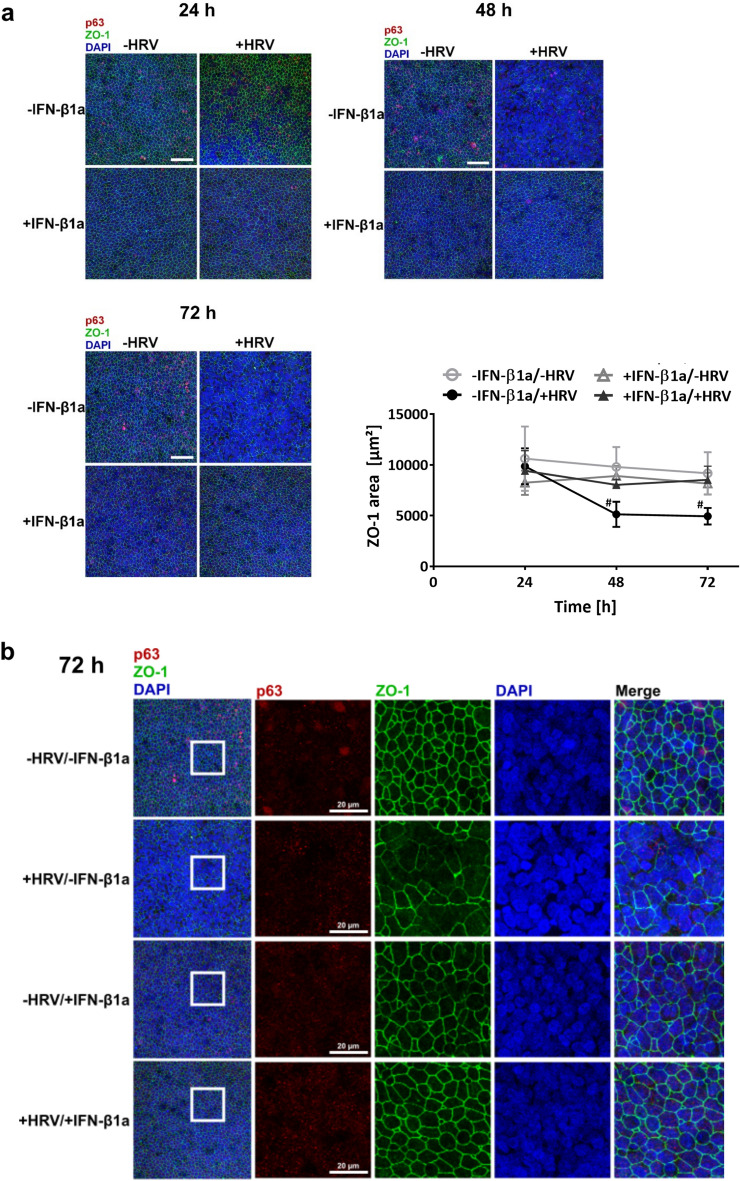
Effect of IFN-β1a pre-treatment and HRV 16 infection on ZO-1 positive TJ of differentiated pBECs in ALI culture. (**a**) Visualization of TJs in ALI cultures after IFN-β1a pre-treatment and HRV 16 infection, immunofluorescence staining. Z-projection images were generated using confocal images. Green: ZO-1, red: p63; scale bar: 50 µm. Figure shows representative images of four independent experiments. Bottom right panel: Quantification of ZO-1 area. n = 4, data represent mean ± SD, # -IFN-β1a/ + HRV vs. -IFN-β1a/-HRV; # p ≤ 0.05. Three-Way ANOVA, Tukey’s Multiple Comparison Test, matching factor: time after infection. (**b**) Close up of insets of immunofluorescence stainings shown in 2a (72 h after viral infection). Corresponding magnification of images for earlier time points are shown in supplementary figure S4a and b. Scale bar 20 µm.

To visualize TJs, cell layers were stained for ZO-1 using fluorescently labeled antibodies and analyzed using CLSM. ZO-1 positive areas were visible as net-like structures throughout the cell layer in uninfected cultures (Fig. 2a, see figure S3a for respective isotype control stainings), and magnification revealed distinct localization of ZO-1 positive areas at cell–cell junctions (Fig. 2b and S4). After 48 h and 72 h of infection, HRV 16 infection had led to a marked disruption of these structures, which could be entirely prevented by pre-treatment with IFN-β1a.

To quantify these effects, the contiguous area staining positive for ZO-1 was computed. The ZO-1 area of untreated, HRV 16-infected cells was 44.39 ± 22.49% smaller after 48 h, and 44.02 ± 14.42% smaller after 72 h post viral infection compared to that of untreated, uninfected cells (48 h: -IFN-β1a/ + HRV 5130 ± 1235 µm^2^ vs. -IFN-β1a/-HRV 9794 ± 1965 µm^2^, p = 0.0148; 72 h: -IFN-β1a/ + HRV 4939 ± 810 µm^2^ vs. -IFN-β1a/-HRV 9177 ± 2075 µm^2^, p = 0.0376) (Fig. 2a, bottom right panel). The ZO-1 positive areas of IFN-β1a pre-treated cells, with or without HRV 16 infection, were similar to that of untreated, uninfected cells, showing that pre-treatment with IFN-β1a can protect TJs from HRV 16-inflicted damage.

In order to visualize basal epithelial cells in the multilayered model, cultures were stained additionally for the basal cell marker p63. We did not observe a consistent change in p63 intensity or number of p63 positive cells in relation to the treatment.

To determine if the infection-associated disruptions of ZO-1 positive TJs were linked to altered expression of genes relevant to the formation of tight and adherence junctions, we next determined mRNA expression of the TJ components ZO-1 and claudin-4, as well as of the adherence junction protein E-cadherin.

No significant effects of viral infection on mRNA levels of the selected genes were measured at any time point after HRV 16 infection, except for a significantly lower mRNA expression of claudin-4 after 48 h of HRV 16 infection in untreated, HRV 16-infected cells compared to IFN-β1a pre-treated, infected cells (figure S5a-c).

### IFN-β1a pre-treatment protects pBECs in ALI culture from HRV 16-induced damage to cilia

**Fig. 3:**
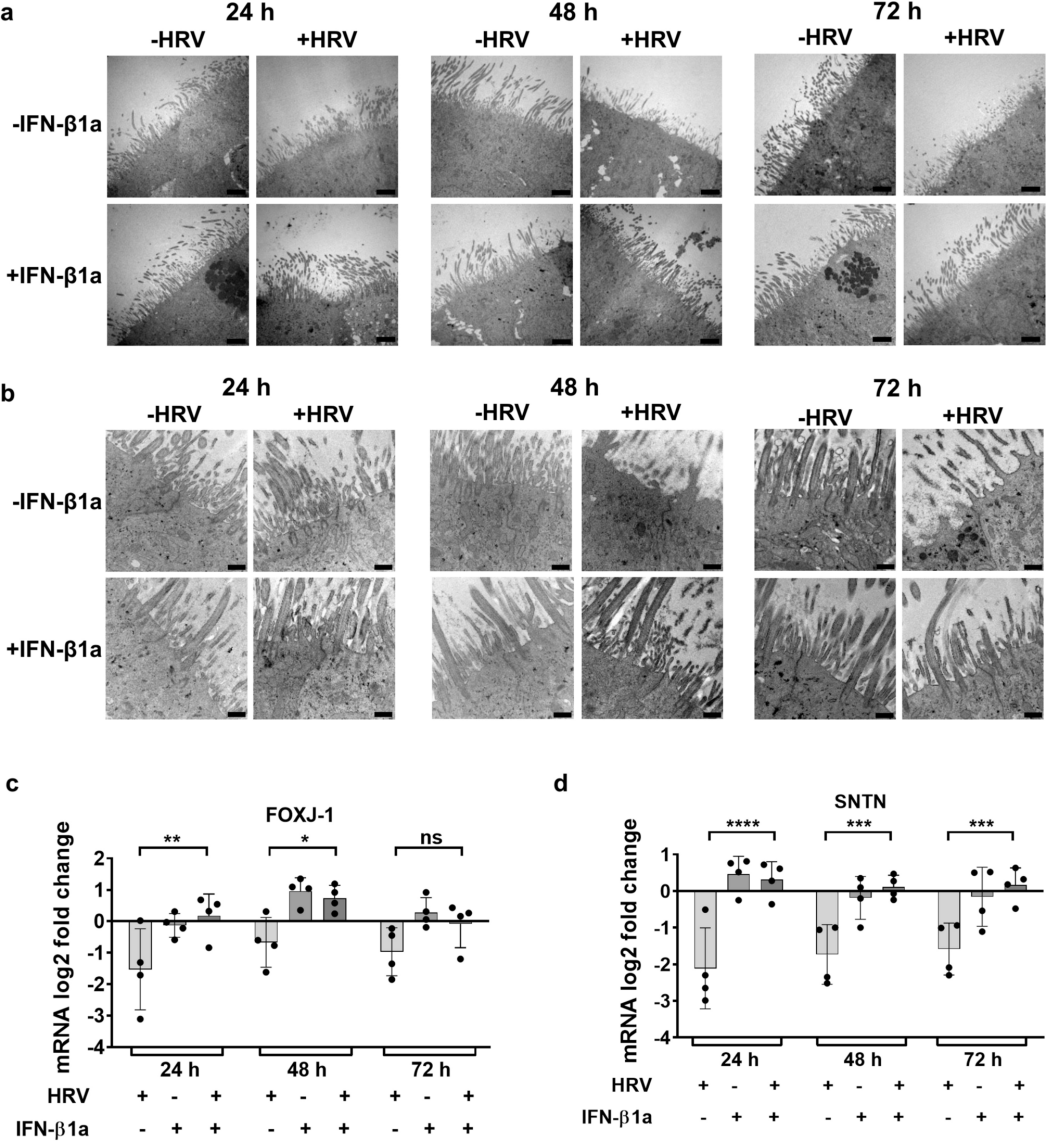
Effect of HRV 16 infection and IFN-β1a pre-treatment on ciliated bronchial epithelial cells. (**a**) Electron microscopy pictures of cilia structure 24 h, 48 h and 72 h after HRV 16 infection in untreated and IFN-β1a pre-treated cells, (**a**) 5000 × magnification. Scale bar: 2500 nm; (**b**) 25,000 × magnification, scale bar 500 nm; samples were collected at different time points of one experiment. (c-d) mRNA expression of (**c**) Forkhead Box J1(FOXJ-1) and (**d**) Sentan (SNTN) were assessed by RT qPCR, normalized to GAPDH and mRNA log_2_(fold change) was calculated over uninfected and untreated controls. n = 4, data represent mean ± SD, * p ≤ 0.05 *** p ≤ 0.01; *** p ≤ 0.001, **** p ≤ 0.0001, ns = not significant. Two-Way ANOVA, Tukey’s Multiple Comparison Test.

To assess the effect of HRV 16 infection and IFN-β treatment on ciliated cells, cells were analyzed by electron microscopy. Electron microscopy indicated that HRV 16-infected cultures have shortened cilia and more unciliated cells in comparison to uninfected cells 48 h and 72 h after infection. In contrast, in cells pre-treated with IFN-β1a, HRV 16 infection had no effect on the integrity of the cilia at any time point tested (Fig. 3a and b). Despite a decrease in ZO-1 positive area in virus infected cells at 48h and 72h after viral infection (Fig. 2a), close up of cell–cell junctions in electron micrographs showed morphologically intact tight junction complexes (Fig. 3b).

To further characterize the impact on ciliated cells, mRNA expression of marker proteins for ciliated cells, Forkhead Box J1 (FOXJ-1) and Sentan (SNTN, Cilia Apical Structure Protein) were determined. In the absence of IFN-β1a pre-treatment, HRV 16 infection led to significantly lower expression of FOXJ-1 after 24 h and 48 h when compared to pre-treated cells (mRNA-Expression FOXJ-1, log_2_(fold change), 24 h: -IFN-β1a/ + HRV -1.53 ± 1.29 vs. + IFN-β1a/ + HRV 0.18 ± 0.70, p = 0.0015; 48 h: -IFN-β1a/ + HRV -0.67 ± 0.79 vs. + IFN-β1a/ + HRV 0.73 ± 0.41, p = 0.0125) (Fig. 3c). Similar results were obtained for mRNA expression of SNTN (mRNA-Expression SNTN, log_2_(fold change), 24 h: -IFN-β1a/ + HRV -2.11 ± 1.10 vs. + IFN-β1a/ + HRV 0.31 ± 0.49, p < 0.0001; 48 h: -IFN-β1a/ + HRV -1.73 ± 0.82 vs. + IFN-β1a/ + HRV 0.11 ± 0.32, p = 0.0004; 72 h: -IFN-β1a/ + HRV -1.59 ± 0.16 vs. + IFN-β1a/ + HRV 0.16 ± 0.47, p = 0.0008) (Fig. 3d).

Taken together, these data show that IFN-β1a pre-treatment protects cilia structures from HRV-induced damage, and counteracts the reduced mRNA expression of FOXJ1 and SNTN associated with HRV infection.

### IFN-β1a pre-treatment protects pBECs in ALI culture from HRV-induced decrease in TEER and increase in barrier permeability

HRV 16-induced effects on cells and TJs could negatively affect the permeability and thus the protection against secondary bacterial infections. Therefore, we next assessed the effects of HRV 16 infection and IFN-β1a pre-treatment on a functional level.

**Fig. 4:**
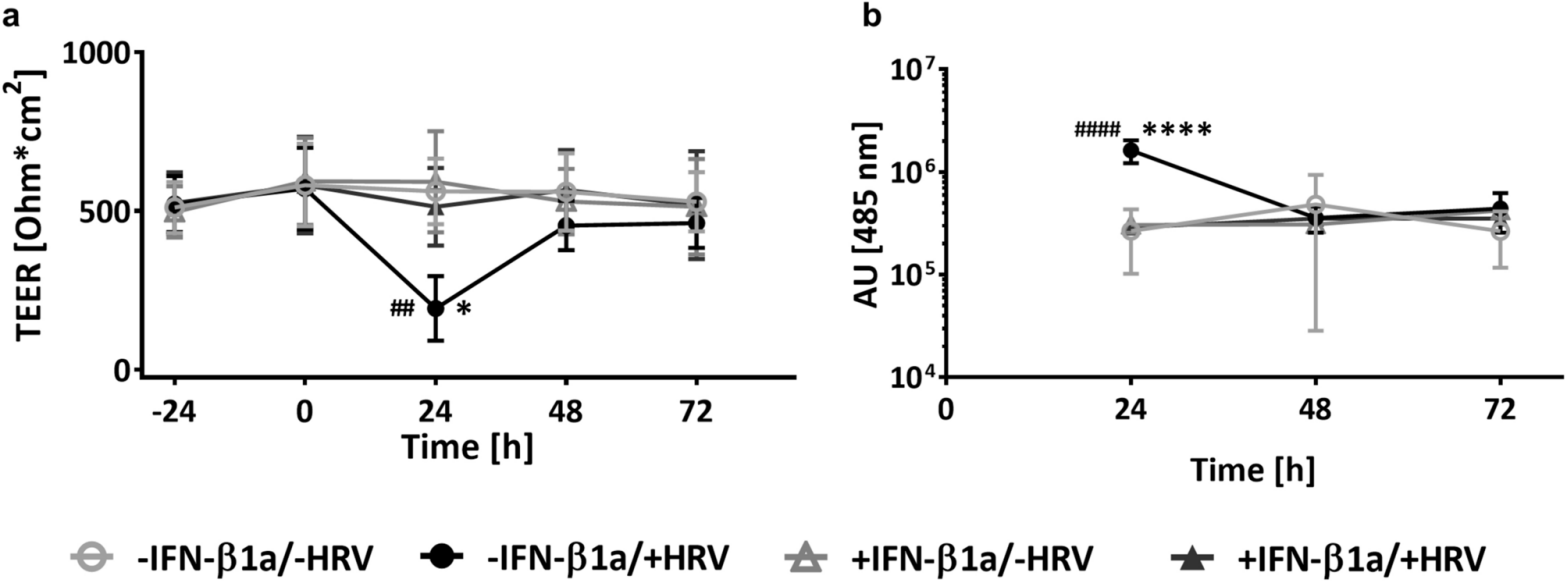
Barrier function of pBECs in ALI culture pre-treated with IFN-β1a and infected with HRV 16. (**a**) TEER measurements of pBECs in ALI culture 24 h before and at viral infection, as well as 24 to 72 h after inoculation with HRV 16. (**b**) Assessment of permeability by measurement of FITC-labelled dextran leakage at 24 to 72 h after HRV 16 infection. n = 4, data represent mean ± SD, * -IFN-β1a/ + HRV vs. + IFN-β1a/ + HRV, # -IFN-β1a/ + HRV vs. -IFN-β1a/-HRV; */# p ≤ 0.05; **/## p ≤ 0.01; ***/### p ≤ 0.001; ****/#### p ≤ 0.0001. Three-Way ANOVA, Tukey’s Multiple Comparison Test, matching factor: time after infection.

TEER of HRV 16-infected cultures was 62.08 ± 27.36% less 24 h after infection when compared to uninfected control cells, and likewise was significantly lower when compared to IFN-β1a pre-treated, HRV 16-infected cells (24 h: -IFN-β1a/ + HRV 193 ± 102 Ohm*cm^2^ vs. -IFN-β1a/-HRV 562 ± 104 Ohm*cm^2^, p = 0.0063 and vs. + IFN-β1a/ + HRV 513 ± 122 Ohm*cm^2^, p = 0.0362) (Fig. 4a). Unexpectedly, we observed TEER levels had returned to levels comparable to those of untreated, uninfected control cells 48 h after infection, and subsequently remained stable up to 72 h after infection.

As a measure for permeability of the epithelial layer, we determined leakage of FITC-labeled dextran (molecular weight 3–5 kDa) from the apical compartment into the basolateral chamber. In line with the results of the TEER measurements, permeability of untreated, HRV 16-infected cell layers was highest 24 h after infection, and returned to the level of untreated, uninfected controls 48 h and 72 h after infection (Fig. 4b). IFN-β1a pre-treatment protected the cells from HRV-induced reduction of TEER and increase in permeability after 24 h of viral infection. TEER values and permeability of IFN-β1a pre-treated, HRV 16-infected cell layers were similar to the results of untreated, uninfected control cells at all time points assessed.

### IFN-β1a pre-treatment reduces bacterial penetration into the cell layer of pBECs in ALI culture

To examine whether IFN-β1a pre-treatment affects bacterial translocation in virus-infected cell layers, we performed viral-bacterial co-infections. To this end, pBECs in ALI culture were initially infected with HRV 16 for 24 to 72 h and subsequently infected with *S. pneumoniae* for 8 h. IFN-β1a treatment and HRV-infection was performed as described above.

**Fig. 5:**
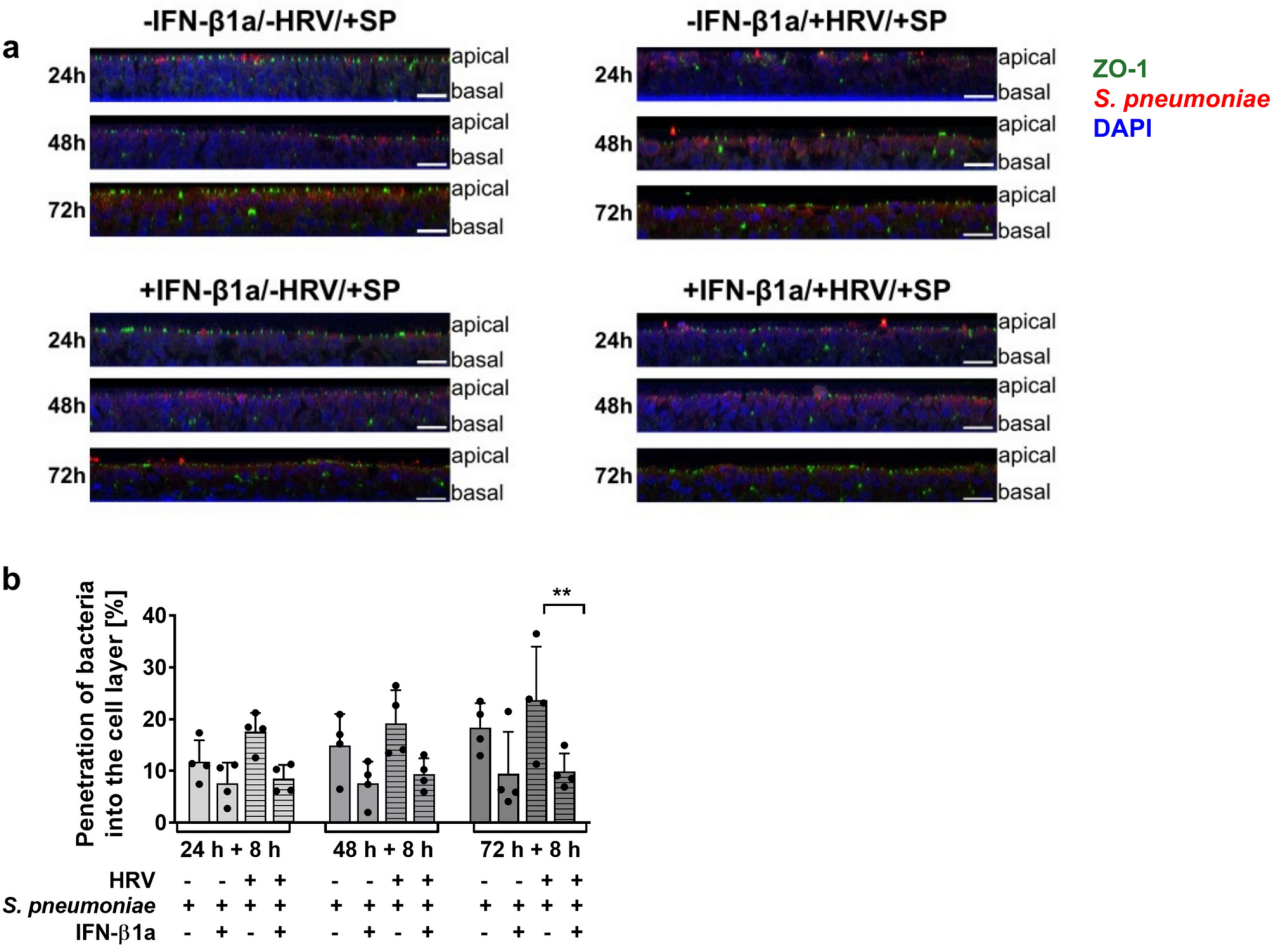
Bacterial translocation during HRV 16-*S. pneumoniae* co-infection into the cell layer of pBECs in ALI culture pre-treated with IFN-β1a. (**a**) Representative orthogonal views of pBECs in ALI culture after 24 h IFN-β1a pre-treatment and 24 h + 8 h, 48 h + 8 h and 72 h + 8 h co-infection with HRV 16 and *S. pneumoniae* (SP), orthogonal views were generated using confocal images. Green: ZO-1, red: *S. pneumoniae*, blue: DAPI; A: apical side, B: basal side; scale bar: 20 µm. (**b**) Quantification of bacterial translocation into the cell layer, expressed as depth in % of average cell layer height. n = 4, data represent mean ± SD, * -IFN-β1a/ + HRV/ + *S. pneumoniae* vs. + IFN-β1a/ + HRV/ + *S. pneumoniae*, ** p ≤ 0.01. Two-Way ANOVA, Tukey’s Multiple Comparison Test.

Translocation of *S. pneumoniae* was visualized using immunostaining and confocal laser scanning microscopy (Fig. 5a). Bacteria migrated further into the cell layer of untreated, co-infected cells than into that of IFN-β1a pre-treated, co-infected cultures or cultures not infected with HRV 16. Bacterial translocation was more pronounced after longer viral infections, with translocation being highest when cells were inoculated with *S. pneumoniae* after 72 h of HRV 16 infection. In contrast, in IFN-β1a pre-treated, co-infected cells, bacterial translocation appeared to be reduced and *S.* *pneumoniae* was mainly identified at the apical barrier at all time points. Quantification of depth of bacterial penetration into the cell layer in orthogonal views confirmed these observations (Fig. 5b). IFN-β1a pre-treatment reduced bacterial translocation of *S. pneumoniae* into the cell layer, with the effect being statistically significant when cells were inoculated with *S. pneumoniae* 72 h after viral infection (depth of penetration of *S. pneumoniae* (SP) into the cell layer, 72 h: -IFN-β1a/ + HRV/ + SP 23.69 ± 10.28% vs. + IFN-β1a/ + HRV/ + SP 9.88 ± 3.2% of average cell layer height, p = 0.0058). Also, a trend towards reduced bacterial translocation was seen in IFN-β1a pre-treated cells within the absence of viral infection (depth of penetration of *S. pneumoniae* into the cell layer, 24 h: -IFN-β1a/-HRV/ + SP 11.81 ± 4.11% vs. + IFN-β1a/-HRV/ + SP 7.63 ± 3.99% of average cell layer height, p = 0.1087; 48 h: -IFN-β1a/-HRV/ + SP 14.91 ± 6.09% vs. + IFN-β1a/-HRV/ + SP 7.61 ± 4.17% of average cell layer height, p = 0.0739). These findings show that IFN-β1a pre-treatment is able to reduce bacterial invasion into the cell layer and can show beneficial and protective effects to the barrier function of differentiated airway epithelial cells.

## Discussion

Type I IFNs have long been known as potent antiviral mediators, and activity against respiratory viruses has been demonstrated previously in various *in vitro* models^33–35,45^. More recently, these findings have prompted the development of inhalation formulations for the treatment of virus-associated asthma exacerbations and COVID-19^25,37^. Several respiratory viruses have been shown previously to disrupt epithelial barrier integrity, which in turn may facilitate bacterial superinfections^28,30,31,46,47^. Thus far, it is unclear whether exogenous type I IFNs are also able to prevent barrier dysfunction associated with viral infections. Using differentiated primary bronchial epithelial cells, we found that pre-treatment with IFN-β1a not only effectively reduced HRV load, but also prevented virus-induced barrier disruption and, during bacterial co-infection, reduced penetration of bacteria into the cell layer.

In line with previous findings^28,48^, infection with HRV 16 progressively reduced cell viability over time in our model, indicated by increasing levels of LDH in apical wash fluid and increased numbers of dead cells stained in LIVE/DEAD assay. IFN-β1a pre-treatment was able to entirely abrogate this virus-induced cytopathic effect.

We next characterized the effects of HRV 16 infection on epithelial barrier function on multiple levels, including quantification of ZO-1 positive TJ complexes, TEER, permeability to fluorescently labeled dextran and bacteria, and integrity of ciliated cells. HRV 16 infection impaired the integrity of the epithelial barrier in all of these readouts. Staining for ZO-1 revealed a loss of ZO-1 positive TJ complexes, which worsened progressively over time. Disruption of TJs following HRV infection has been shown in previous studies, with varying degrees of intensity depending on the strains used^28,30,31^. Here, we show additionally that pre-treatment with IFN-β1a can entirely protect the epithelial cell layer from this virus-inflicted damage.

The disruption of ZO-1 positive TJ complexes could either be caused by degradation, impaired production of this protein or by translocation to other cellular compartments. Gagliardi et al. observed disrupted TJs after HRV infection. As they were unable to detect a reduction in ZO-1 at protein level, the authors concluded that there was a translocation of the tight junction proteins^28^. Of note, electron micrographs in our experiments showed morphologically intact TJ, despite loss of ZO-1 localization at cell–cell junctions and clear functional loss of barrier integrity as shown by increased permeability of the cell layer and (transient) drop in TEER. These observations might reflect a discrepancy between morphological and functional integrity of the tight junction complexes. Partially redundant functions have been described for multiple TJ proteins^49–51^, and the discrepancy between loss of ZO-1 positive TJ area and morphologically intact TJ complexes in electron microscopy might be related to a partial, and functionally insufficient, compensation of loss of ZO-1 localization by other tight junction proteins.

In addition to TJ complexes, also ciliated cells were severely affected by virus infection, and damage to cilia similarly worsened progressively over time. On mRNA level, markers of ciliated cells were likewise reduced. These observations confirm earlier findings, which showed that HRV infection can damage ciliated cells and impair ciliary function^28,44,52,53^. Also on the level of ciliated cells, pre-treatment with IFN-β1a protected cells from virus-induced damage.

As additional indicators of barrier integrity, we measured TEER and permeability of cell layers to FITC-labeled dextran. HRV infection decreased TEER and increased permeability to FITC-labeled dextran 24 h after infection in untreated, but not in IFN-β1a pre-treated cells. Of note, the virus-induced effects on these readouts were transient and had recovered by 48 h after infection, whereas dissociation of TJs, damage to ciliated cells and permeability to bacteria worsened progressively over time. As mentioned above, previous reports have indicated that some TJ proteins can partially compensate for loss of function of related proteins^49,50^. Such compensation mechanisms could explain why initial drop in TEER and increase in permeability to small molecules recover over time, whereas other indicators of malfunction of the epithelial barrier are sustained or even deteriorate. Importantly, all signs of virus-associated damage to epithelial barrier function, regardless of their temporal course, could be prevented by pre-treatment with IFN-β1a in our model.

Key inflammatory mediators induced by infection with HRV 16 were lower in IFN-β1a treated cells than in untreated cells, suggesting that IFN-β1a treatment might also be able to curb virus-associated excessive inflammation. In contrast to observations made in HRV 1B-infected epithelial cells of asthma patients^33^, IFN-β1a treatment did not lead to a significant reduction of virus-induced IP-10 in our model. This could be explained by the induction of IP-10 by IFNs and likely represents a direct effect of the IFN-β1a treatment^54^.

Viral infections of the epithelium of the lower respiratory tract increase the likelihood of bacterial superinfections, which in turn are associated with increased mortality^55,56^. Therefore, we next sought to further assess the functional consequences of impaired barrier function in our model. To this end, we conducted bacterial superinfections with *S. pneumoniae* at various time points after viral infection and analyzed the depth of penetration of bacteria into the epithelial layer. The most pronounced invasion into the cell layer was seen when cells were infected with bacteria 72 h after viral infection. These findings indicate that susceptibility to invasive bacterial infection, unlike permeability to FITC-labeled dextran, does not revert to baseline over time. Again, IFN-β1a was able to prevent virus-induced damage, and also in the absence of viral infection, pre-treatment tended to cause less pronounced bacterial invasion. The latter is mirroring observations in immortalized bronchial epithelial cells, which showed reduced expression of the *S. pneumoniae* adhesion receptor PAFR^57^. However, the role of type I IFNs during bacterial infection is ambiguous, and both, beneficial^58,59^ as well as negative^60,61^ effects on antibacterial immunity have been described in the past. Additional analyses will therefore need to consider species-specific effects, as well as the effects of immune cells in more complex models.

Taken together, our results show that IFN-β1a not only exhibits potent antiviral activity against HRV 16, but can also protect the airway epithelium from virus-induced barrier dysfunction. Thereby it might provide additional benefits in the treatment of respiratory tract infections with HRV and other viruses, and potentially reduce the risk of bacterial superinfections. The broad spectrum of viruses against which type I IFNs can act moreover makes them attractive candidates for a treatment against emerging viruses, and viruses against which vaccinations or targeted antiviral therapies are missing. During the SARS-CoV2 pandemic, type I IFNs have been shown to be effective against the virus *in vitro*^62^ and a phase 3 clinical trial in patients hospitalized due to COVID19 has been completed in 2023. Although this trial did not meet its primary endpoint, post hoc analysis did show a benefit of IFN treatment when administered on top of routine care in certain high risk sub-groups^37^. Preliminary results also indicate a reduction of relative risks to develop long COVID symptoms^63^.

The protective effect of IFN-β1a against virus-associated dysfunction of the epithelial barrier might be a consequence of reduced viral load, and further work is needed in order to clarify the underlying mechanism. While cell death due to viral infection certainly contributes to damage of the epithelial layer, increased permeability can be seen already early on during infection, and even in the absence of virus-induced cytopathic effect^30,64^.

The underlying mechanism of HRV-induced increase in epithelial permeability remains to be identified. Additionally, future works should address the role of immune cells, as well as the efficacy of treatment once infection has been established. Moreover, synergistic effects of type I and III IFNs could be leveraged to improve IFN-based treatment strategies^65^. With potent and broad-acting antiviral effects, IFN-based formulations hold promise for treatment and prevention of respiratory virus infections in individuals at risk for a severe course of such events, e.g., patients with underlying chronic respiratory diseases.

## Supplementary Information

Supplementary Information.

## Data Availability

The original data presented in the study are included in the article and supplementary material. Further inquiries can be directed to the corresponding author.
