# Supplementary material for: Protective effect of interferon type I on barrier function of human airway epithelium during rhinovirus infections in vitro
Source: Sci Rep. 2024 Dec 16;14:30510. doi: 10.1038/s41598-024-82516-2 (PMC11649702; doi:10.1038/s41598-024-82516-2)
Supplement: Supplementary file 1 — Supplementary Information. [file 41598_2024_82516_MOESM1_ESM.docx]

# Supplemental Material

# Supplemental Methods

## Overview of infection and stimulation protocol


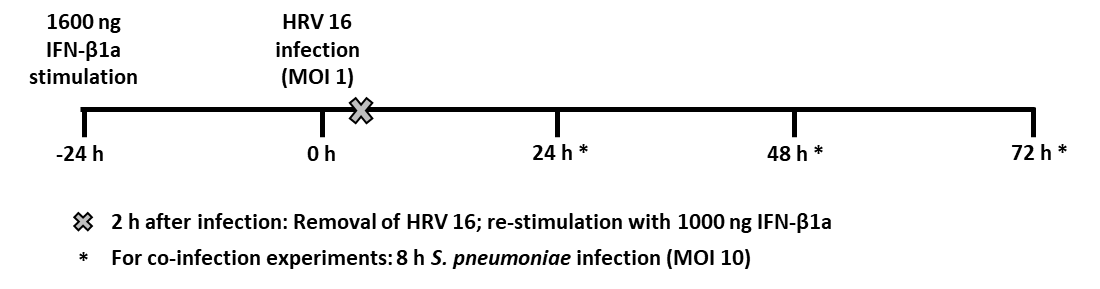


**Figure S1: Schematic timeline of experiments.** ALI cultures were stimulated for 24 h with IFN-β1a before cells were infected with HRV 16 in IFN-free media. Virus inoculum was removed after 2 h and cells were again stimulated with IFN-β1a and incubated for 24 to 72 h. In case of co-infection, cells were inoculated with *S. pneumoniae* for 8 h at the indicated time points.

## Cytometric Bead Array (CBA)

Concentrations of the cytokines IL-6, IL-8, IP-10, and TNF-α in basal medium were analyzed using the BD™ CBA Flex Set System (BD Bioscience-PharMingen, San Diego) according to the manufacturer’s instructions. Measurements were performed using a BD FACSVerse™ Cell Analyzer (BD, Franklin Lakes). The lower limit of quantification (LLoQ) for all analytes was 10 pg/mL. Values below the LLoQ were replaced with 0.5 x LLoQ for further analysis.

## RT qPCR assays

Table S1: Primer and probe sequences of RT qPCR assays.

| Target |  | Sequence (5' - 3') |
| --- | --- | --- |
| GAPDH | Forward primer^1^ | ATTCCACCCATGGCAAATTC |
|  | Reverse primer^1^ | CGCTCCTGGAAGATGGTGAT |
|  | Probe^1^ | JOE-CGTTCTCAGCCTTGACGGTGCCA-BHQ1 |
| HRV | Forward primer^2^ | GGTGTGAAGAGCCGCGTG |
|  | Reverse primer^2^ | CAAAGTAGTCGGTCCCATCC |
|  | Probe^2^ | FAM-TCCTCCGGCCCCTGAATGTGG-BHQ1 |
| ZO-1 | Forward primer^3^ | GCGGTCAGAGCCTTCTGATC |
|  | Reverse primer^3^ | CATGCTTTACAGGAGTTGAGACAG |
|  | Probe^3^ | FAM-ACTCGCCGCAGCAGCCAAGCAAT-BHQ1 |
| Claudin-4 | Forward primer | TCTGCTCACACTTGCTGGCT |
|  | Reverse primer | CAGAGTGCACCTTTGCACCG |
|  | Probe | FAM-TGCGCATCAGGACTGGCTTTATCTCC-BHQ1 |
| E-Cadherin | Forward primer^3^ | CCCACCACGTACAAGGGTC |
|  | Reverse primer^3^ | CTGGGGTATTGGGGGCATC |
|  | Probe^3^ | FAM-CGAGGCTAACGTCGTAATCACCACACTGA-BHQ1 |
| FOXJ-1 | Forward primer^4^ | CAACTTCTGCTACTTCCGCC |
|  | Reverse primer^4^ | CGAGGCACTTTGATGAAGC |
|  | Probe | FAM-ACGCAGATCCCACCTGGCAGAA-BHQ1 |
| SNTN | Forward primer | GTCAGTAGCGACCTAGAGCAC |
|  | Reverse primer | GCCTGGCCAATTCATTACTGT |
|  | Probe | FAM-CATCTACTTGATGGAACTGCTGAGGA-BHQ1 |

all primers and probes were obtained from Sigma Aldrich, Taufkirchen

Where indicated, primer/probe sequences were taken from the following sources: ^1^ [1]; ^2^ [2]; ^3^ [3]; ^4^ [4]. All other assays/oligonucleotides were designed in-house.

## Isotype control stainings for immunofluorescence

Isotype control stainings were performed using Mouse IgG2 kappa Isotype Control (FITC conjugated, Thermo Fisher Scientific, Waltham, for anti-ZO-1 antibody), Rabbit IgG monoclonal EPR25A Isotype control (abcam, Cambridge (UK), for anti-p63 antibody) or Rabbit IgG Isotype Control (polyclonal, Thermo Fisher Scientific, Waltham, for anti-S. pneumoniae antibody) instead of the respective primary antibody. Preparation of specimens, antibody concentrations, incubation conditions, and (if applicable) secondary antibodies were identical to the ones used for target-specific antibodies.

# Supplemental data


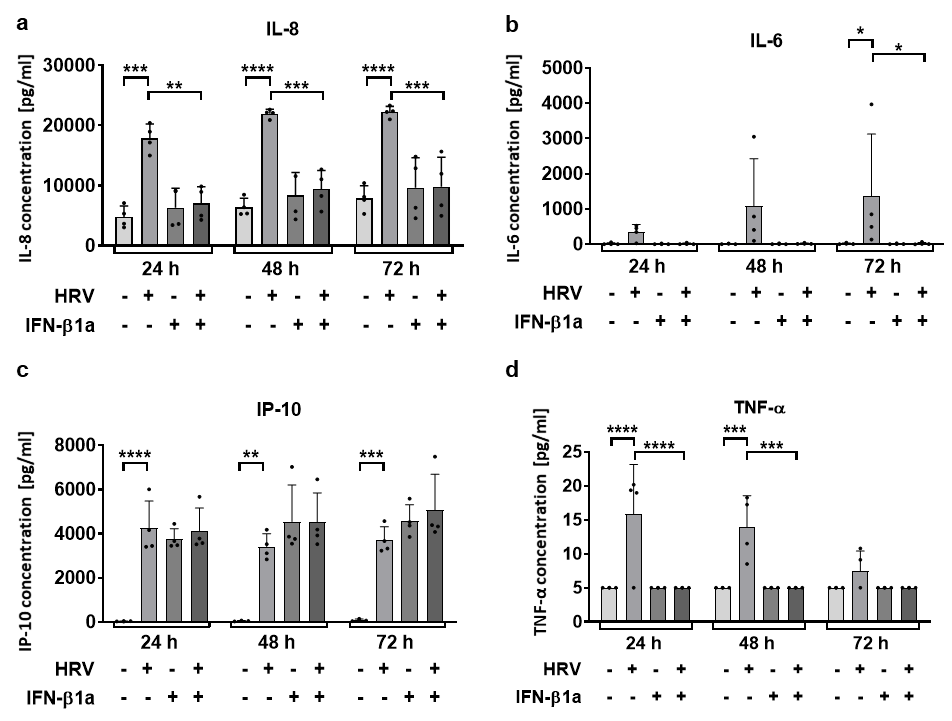


**Figure S2**: Cytokine concentrations in basal media of pBECs in ALI culture after 24 h IFN-β1a pre-treatment and 24 h to 72 h HRV 16 infection. Concentration of (a) IL-8, (b) IL-6, (c) IP-10 and (d) TNF-α were assessed in basal media by CBA. LLoQ = 10 pg/mL, all values below the limit were replaced with 0.5*10 pg/mL. n=4, data represent mean ± SD, * p≤0.05; ** p≤0.01; *** p≤0.001; **** p≤0.0001. Two-Way ANOVA, Tukey's Multiple Comparison Test.


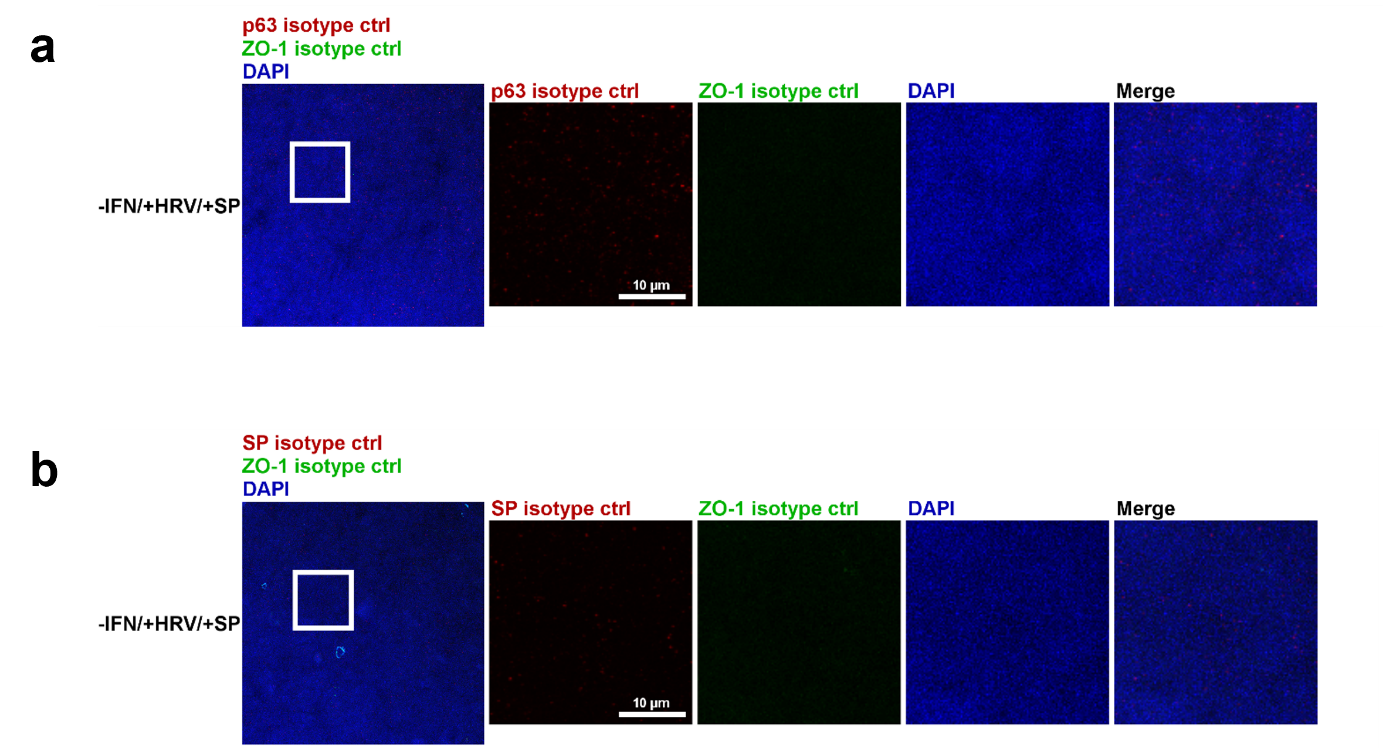


**Figure S3**: Isotype control stainings for immunofluorescence-based detection of ZO-1, p63 and *S. pneumoniae*. (a) Immunofluorescence staining using isotype control antibodies for anti-ZO-1 and anti-p63 antibodies; (b) Immunofluorescence staining using isotype control antibodies for anti-ZO-1 and anti*-S. pneumoniae* antibodies


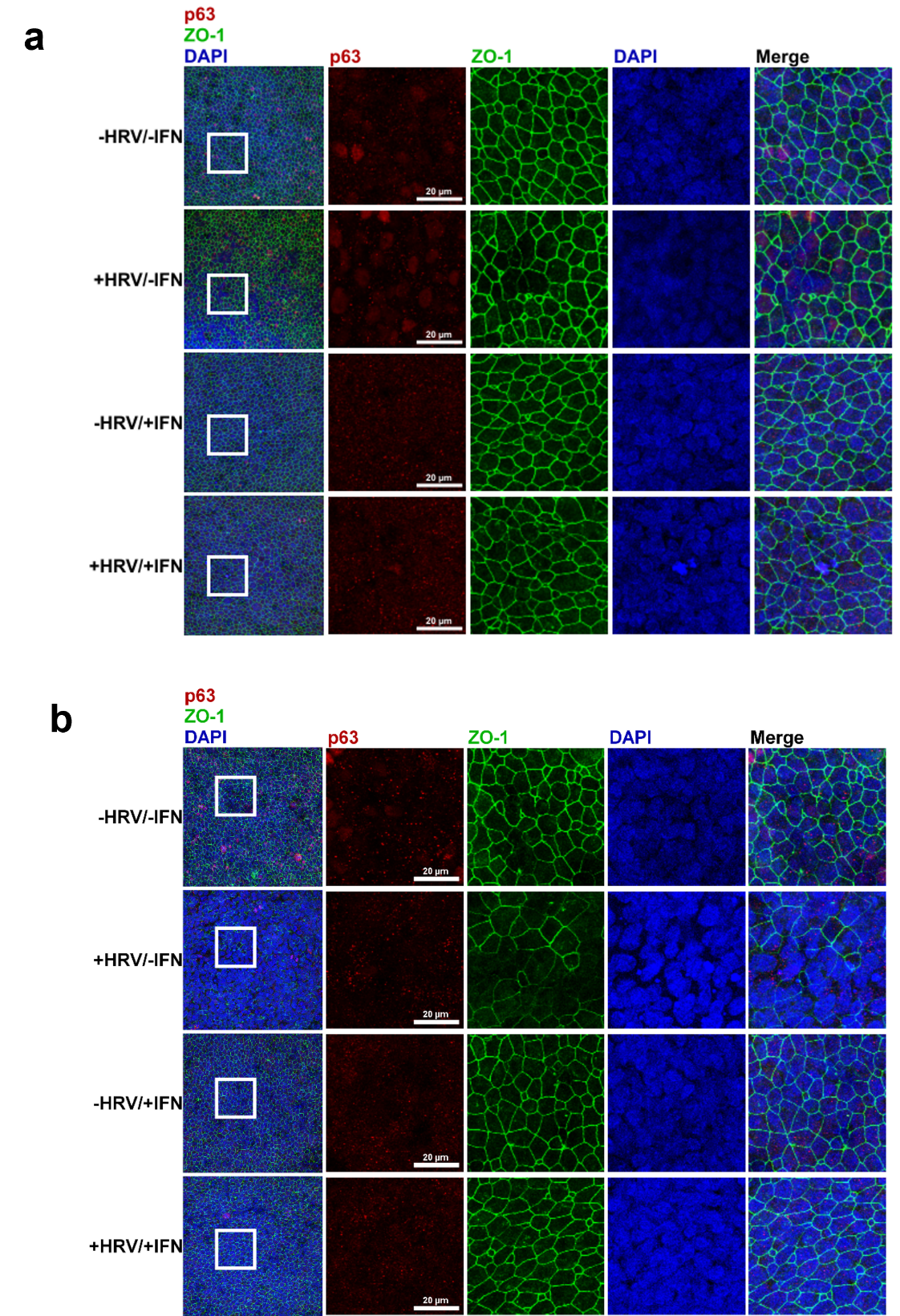


**Figure S4:** Close up of insets of immunofluorescence stainings shown in 2a (a) 24h after viral infection; (b) 48h after viral infection. Scale bar represents 20 µm.


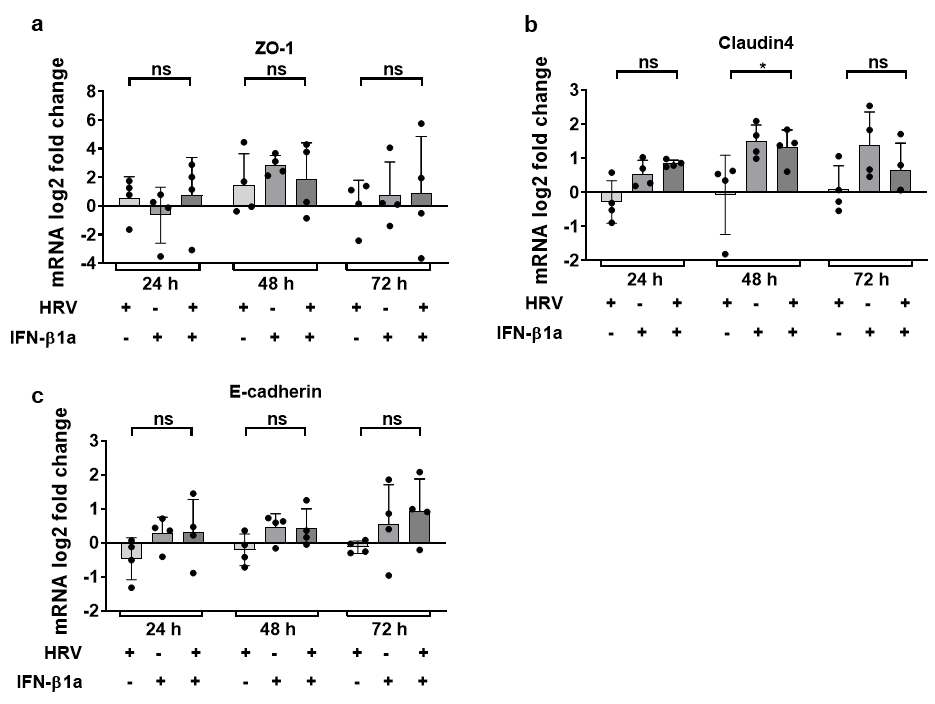


**Figure S5**: mRNA expression of tight and adherence junction genes in pBECs in ALI culture after 24 h IFN-β1a pre-treatment and 24 h to 72 h HRV 16 infection. Relative change of (a) ZO-1, (b) Claudin-4 and (c) E-cadherin mRNA expression was assessed in cell lysates by RT qPCR, normalized to GAPDH and mRNA log_2_(fold change) was calculated over untreated, uninfected control cells. n=4, data represent mean ± SD, * p≤0.05. Two-Way ANOVA, Tukey's Multiple Comparison Test.

# References

1. Hillyer P, Shepard R, Uehling M, Krenz M, Sheikh F, Thayer KR, Huang L, Yan L, Panda D, Luongo C, Buchholz UJ, Collins PL, Donnelly RP and Rabin RL (2018) Differential Responses by Human Respiratory Epithelial Cell Lines to Respiratory Syncytial Virus Reflect Distinct Patterns of Infection Control. J Virol 92. doi: 10.1128/JVI.02202-17

2. Hauptmeier BM, Borg I, Rohde G, Anders A, Kronsbein J, Gatermann S, Bufe A, Blum T, Schultze-Werninghaus G and Bauer TT (2010) Viral colonization in intubated patients: initial pathogen pattern and follow-up. Clin Respir J 4:139-46. doi: 10.1111/j.1752-699X.2009.00160.x

3. Stewart CE, Torr EE, Mohd Jamili NH, Bosquillon C and Sayers I (2012) Evaluation of differentiated human bronchial epithelial cell culture systems for asthma research. J Allergy (Cairo) 2012:943982. doi: 10.1155/2012/943982

4. Tadokoro T, Wang Y, Barak LS, Bai Y, Randell SH and Hogan BL (2014) IL-6/STAT3 promotes regeneration of airway ciliated cells from basal stem cells. Proc Natl Acad Sci U S A 111:E3641-9. doi: 10.1073/pnas.1409781111
